# Effect of Molten Pool Spatial Arrangement on Texture Evolution in Pulsed Laser Additive Manufacturing of Inconel 718

**DOI:** 10.3390/ma15093286

**Published:** 2022-05-04

**Authors:** Manping Cheng, Guoyun Luo, Xianfeng Xiao, Lijun Song

**Affiliations:** 1 State Key Laboratory of Advanced Design and Manufacturing for Vehicle Body, Hunan Provincial Key Laboratory of Intelligent Laser Manufacturing, Hunan University, Changsha 410082, China; chengmanping@hnu.edu.cn; 2 School of Mechatronics Engineering, Nanchang University, Jiangxi 330031, China; xxf@ncu.edu.cn

**Keywords:** laser additive manufacturing, lnconel 718, dendrite growth, crystallographic texture

## Abstract

The epitaxial growth of dendrites, which often results in a strong texture, is the most common phenomenon during the laser additive manufacturing process. In this study, the epitaxial growth of dendrites and texture evolution in three directions were studied by changing the z-increment, pulse period, and track offset, respectively. The influence of the molten pool interface on the growth and competition of dendrites is analyzed. Both green grains (<110> // BD) with rotated cube texture in the molten pool overlapping zones and red grains (<100> // BD) with fiber texture in the molten pool center zones coexist for different z-increment samples, forming the typical sandwich texture feature. In a short pulse period, the dendrites can grow directly epitaxially and form the strong fiber texture due to gentle interface and short distance. With the decrease of the track offset, the molten pool morphology changes from flat to narrow and deep. When θ is close to 90°, dendrites grow along the secondary dendrite arms at the overlapping zone, forming V-shape grains. This work also provides a promising method for texture customization for laser additive manufacturing.

## 1. Introduction

Laser additive manufacturing (LAM) has been widely studied in recent years [1,2]. The texture is a common phenomenon in LAM parts due to unidirectional heat flux and high temperature gradients, which can affect the performance [3]. However, it is a great challenge to achieve a controllable texture of the LAM parts, due to the numerous parameters of the LAM process. Therefore, tremendous work has been conducted to study dendrite growth and texture evolution [4,5,6,7].

The growth of dendrites is affected by the solidification parameters. Gäumann et al. [8] established the relationship between the temperature gradient (G), solidification velocity (V), and volume fraction of equiaxed grains (ø) to illustrate the phenomenon of columnar to equiaxed transition (CET) during the LAM process. It is important to get accurate solidification parameters (G, V) to predict microstructure morphology. Wang et al. [9,10] established the 3D heat transfer model considering heat conduction or heat convection. They obtained the solidification parameters (G, V) through the model and studied the effect of different substrate orientations on the CET. Liu et al. [11,12,13] established a complex three-dimensional mathematical model with temperature field, flow field, and molten pool morphology for the powder-feeding LAM process. Based on this model, the effect of substrate crystallographic orientations and processing parameters (laser power, scanning speed, powder feeding rate, and inclination of coaxial nozzle) on crystal growth were studied. These works show that the CET model has good applicability on single-crystal substrate. It is worth noting that the CET model explains the transition from columnar to equiaxed in a single molten pool. Due to the high remelting rate, the equiaxed grain in the top of molten pool will be remelted, and the LAM process is performed as epitaxial growth of dendrites [14]. The dendrites with different orientations will compete [15]. The interdendritic competitive growth is affected by the direction of the temperature gradient, which is also the direction of heat flux. The direction of heat flux is also the normal direction of the molten pool interface; thus, the interdendritic competitive growth is directly related to the molten pool morphology.

In recent years, researchers have paid attention to the relationship between molten pool morphology and texture. Garibaldi et al. [16] found that the texture changes from fiber to cubic texture when the molten pool morphology changes from shallow to deep. Sun et al. [17] found that the molten pool morphology and texture could be controlled by the laser energy density, where a parabolic shaped molten bead resulted in crystallographic lamellar microstructure under lower energy density, while a near keyhole shape led to a single-crystalline-like texture under higher energy density. Gokcekaya et al. [18] tailored the texture by adjusting the molten pool morphology by laser energy density in a laser powder bed fusion of Inconel 718. They found the texture features with a sandwich structure, which can improve mechanical performance. Andreau et al. [19] revealed that the texture is associated with the variations in molten pool morphology. McLouth et al. [20] studied the effect of laser focus shift on the microstructure and texture. They found that the coarse dendrites and strong texture were formed at the shifted plane, while the equiaxed microstructure and weak texture were obtained at the focal plane. The formation of strong texture is related to the gentle molten pool morphology formed. It is worth noting that the above studies did not consider the effect of the molten pool spatial arrangement on the texture.

The pulsed LAM offers many advantages in modulating microstructure [21,22]. The parts are formed by the accumulation of a single molten pool for the pulsed LAM process. Competition between dendrites in different molten pools affects dendrite growth. Therefore, the spatial arrangement of the molten pool will also affect the evolution of texture. In fact, the arrangement of molten pools in three-dimensional space is related to the overlapping rate of molten pools in the three directions of build direction (BD), scanning direction (SD), and track offset direction (TD). Nenadl et al. [23] reported grain growth at different track offsets. However, there is still a lack of systematic research on the evolution of texture in three directions under pulsed LAM.

The objective of this work is to systematically research the epitaxial growth of dendrites and texture evolution in three-dimensional space by changing the z-increment, pulse period, and track offset, respectively. The growth and competition of dendrites at the molten pool interface is discussed. This work manifests a promising method for texture customization by manipulating the molten pool spatial arrangement for laser additive manufacturing.

## 2. Materials and Methods

**Figure 1:**
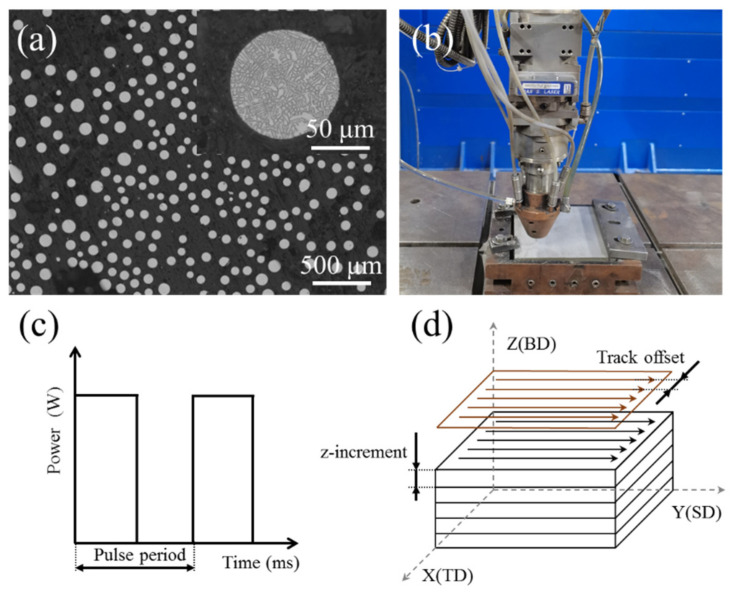
(**a**) Morphology of Inconel 718 alloy powder. (**b**) Experimental setup. (**c**) Schematic diagram of pulsed laser. (**d**) Scanning strategy.

The Plasma-Rotating Electrode Process (PREP) Inconel 718 powders, with nominal chemical compositions (wt%) of Mo 3.1, Nb 5.1, Cr 18.39, Ni 52.67, Al 0.46, Ti 0.76, and Fe balance, were used as the deposit material, and 316L stainless steel plate with dimensions of 120 mm × 120 mm × 9 mm was used as substrate. The Inconel 718 alloy powder used in the experiment has a smooth surface and good sphericity, as shown in Figure 1a. The particle size distribution of the powder was between 45 to 105 μm. The bulk samples were fabricated using a coaxial powder feed LAM system under the unidirectional scanning strategy, as shown in Figure 1d. The change of the scanning strategy caused the change of the heat flux direction. In order to exclude the influence of the scanning strategy on the texture, we chose the unidirectional scanning strategy. The LAM system consists of a fiber laser (IPG YLS-5000, NY, USA) with 5000 W maximum power, a motion control system, a laser cladding head, and a metal powder feeder. The experimental setup is illustrated in Figure 1b. As shown in Figure 1c, the laser was modulated into a square waveform pulse. The experiments were designed to study the competitive growth of dendrites and texture evolution in three-dimensional space (build direction (BD), scanning direction (SD), and track offset direction (TD)). The process parameters are listed in Table 1. Sample A, sample B, and sample C were used to study the build direction (BD); the sample D and sample E were used to study the scanning direction (SD); sample F, sample D, and sample G were used to study the track offset direction (TD). Other constant process parameters were as follows: power of 900 W, duty cycle of 50%, and carrier gas flow of 10 L/min.

After the experiment, the electro-discharge machining was used to cut samples. The samples were grinded with sandpaper, polished, and then etched with a solution of 10 mL H_2_O_2_ + 10 mL HCl + 10 mL H_2_O for optical micrograph (OM). The samples for electron backscattered diffraction (EBSD) testing were prepared by first grinding with 2000-grit sandpaper, then electropolished for 40 s at 20 V in a 10% perchlorate alcohol solution. EBSD data were analyzed using HKL Channel 5 software. In order to better distinguish the center of the molten pool, hardness indentation was produced in the center of the molten pool before the EBSD test.

## 3. Results

### 3.1. Texture Evolution in Build Direction

**Figure 2:**
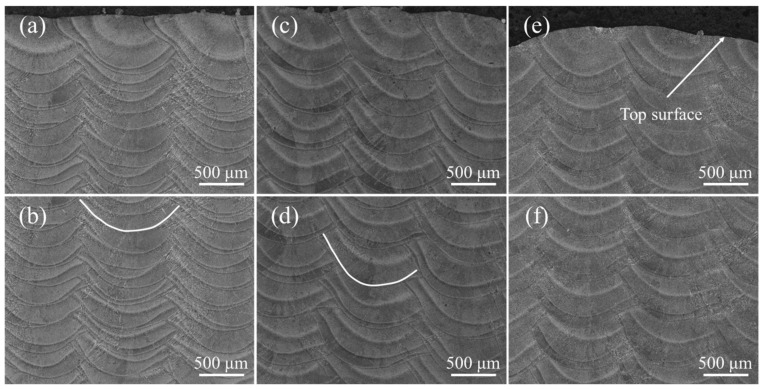
The microstructure with different z-increment: (**a**,**b**) 0.2 mm; (**c**,**d**) 0.4 mm; (**e**,**f**) 0.6 mm.

Figure 2 shows optical morphologies of the transverse section under different z-increment. The molten pool boundary was a semi-circular arc. At z-increment of 0.2 mm, the top surface of the molten pool was flat (Figure 2a). With an increase of the z-increment, the top surface of the molten pool became slightly inclined (Figure 2c,e). This indicates that different z-increment affect the flatness of the deposited samples. The inclination of the molten pool was related to the remelting rate of the molten pool. High z-increment reduced the remelting of the deposited track so it was inclined to the new deposition side. The layer thicknesses under different process parameters were measured. The layer height for z-increment of 0.2 mm, 0.4 mm, and 0.6 mm were 0.21 mm, 0.39 mm, and 0.5 mm, respectively. In all samples, no obvious pores can be seen.

**Figure 3:**
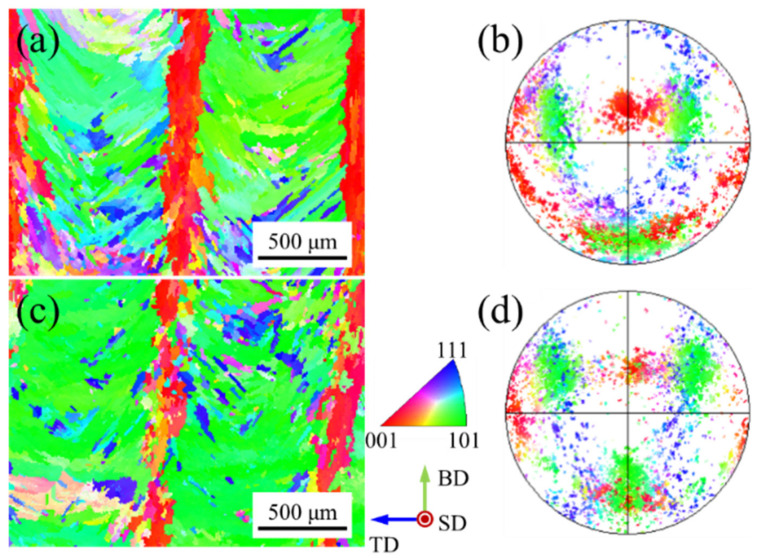
Inverse pole figure (IPF) maps and corresponding pole figures with different z-increment: (**a**,**b**) 0.2 mm; (**c**,**d**) 0.4 mm.

Figure 3 shows the inverse pole figure (IPF) map and corresponding pole figure with different z-increment. The inverse pole figure (IPF) maps show the typical sandwich texture feature (Figure 3a,c). The green grains (<110> // BD) are distributed in the overlapping zones of the molten pool, while the red grains (<100> // BD) are distributed in the center of the molten pool (Figure 3a). The green grains (<110> // BD) and red grains (<100> // BD) exhibit rotated cubic texture and fiber texture at the corresponding pole figure, respectively (Figure 3b). It is worth mentioning that Gokcekaya et al. [18] reported that the sandwich texture appears in the SLM process, and they found that this unique crystallographic texture can improve the mechanical properties of the formed parts. They also mentioned that the sandwich texture feature does not occur in powder-fed LAM. However, the sandwich texture features were obtained in powder-fed pulsed LAM of Inconel 718 alloy due to the arc-shaped molten pool morphology in this work.

The positions of the fixed pole point of the fiber texture are consistent in the pole figure (Figure 3b,d). The dendrites at the center of the molten pool are not affected by the dendrites from the overlapping zones for deep molten pool morphology. The heat flux unidirectionally dissipates at the center of the molten pool interface under the unidirectional scanning path, forming the fiber texture [17]. At z-increment of 0.2 mm, the red grains (<100> // BD) in the center of the molten pool are regular (Figure 3a), while they become discrete at z-increment of 0.4 mm (Figure 3c). The distance between the two molten pool interfaces increases with the increase of layer height, which increases the deviation angle of the heat flux direction between two molten pools, resulting in weak epitaxial growth of dendrites. The grains with other colors are embedded in the green grains (<110> // BD) in the inverse pole figure (IPF) map (Figure 3a,c) and they are scattered in the corresponding pole figure (Figure 3b,d). Since the molten pool boundary is a three-dimensional surface, the direction of heat flux will change during the movement of the solidification interface [24], which increases the probability of the formation of stray grains. At the same time, the pulsed LAM process is multi-directional solidification from the whole envelope of the molten pool [25], forming grains with other directions.

**Figure 4:**
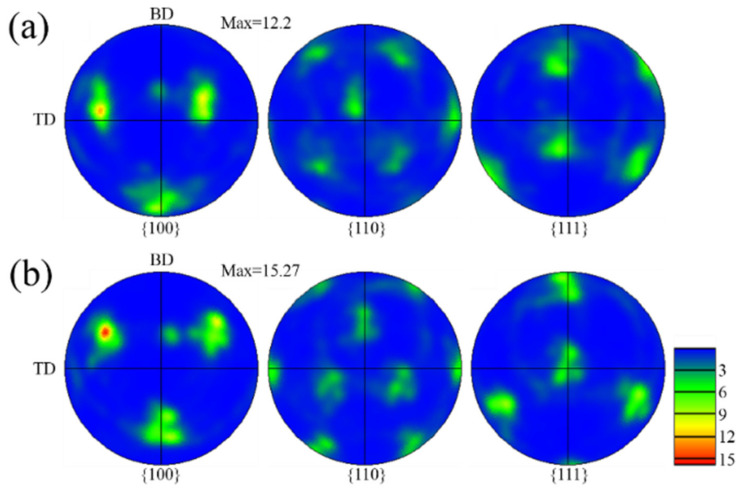
Pole figure with different z-increment: (**a**) 0.2 mm; (**b**) 0.4 mm.

Figure 4 shows the pole figures with different z-increment. The texture characteristics with different z-increment are consistent (Figure 4a,b). The main reason for this is that the pole figures reflect the green grains (<110> // BD) due to the large proportion in the inverse pole figure (IPF) map (Figure 3a,c). Further, in the optical morphologies, the growth of dendrites under the different samples are similar (Figure 2b,d). In the (100) pole figure, the fixed pole point of the fiber texture (red grains (<100> // BD) can also be observed. It is worth mentioning that, compared to the z-increment of 0.2 mm with a maximum multiple of uniform density (MUD)of 12.2 (Figure 4a), the texture intensity is enhanced for the z-increment of 0.4 mm with a maximum MUD of 15.27 (Figure 4b). Compared to the z-increment of 0.4 mm, there are more red grains (<100> // BD) distributed in the inverse pole figure (IPF) map at z-increment 0.2 mm (Figure 3a), which is the main reason for the reduction of texture intensity in the pole figure.

**Figure 5:**
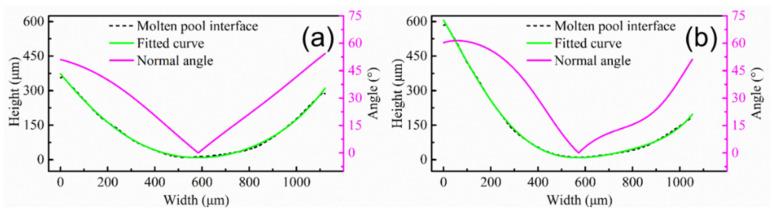
Molten pool interface and normal angle with different z-increment: (**a**) 0.2 mm; (**b**) 0.4 mm.

First, deposition efficiency and formability can be affected by the z-increment for the powder-fed LAM process. The low z-increment can ensure the formability of the part, but it reduces the deposition efficiency. In addition, the molten pool morphology and the remelting ratio of the molten pool can be changed by the z-increment, altering the epitaxial growth of dendrites between different molten pools. The deviation angle of the heat flux direction between different layers is small due to the low layer height for low z-increment, which is beneficial for the epitaxial growth of dendrites at the bottom of the molten pool. It is worth noting that the texture characteristics in the overlapping zone are less affected by the z-increment. In order to further study the characteristics of the molten pool interface, a typical molten pool interface was selected in the optical morphologies marked with the white curve (Figure 2b,d). The molten pool interface was fitted using image processing software to solve the normal angle (the angle between the normal direction of molten pool interface and the Z axis). Figure 5 shows the molten pool interface and normal angle under different z-increment. The molten pool interface and normal angle are symmetrical at z-increment of 0.2 mm, as shown in Figure 5a. The central zone of the molten pool interface is flat, which is the main reason for the formed fiber texture in the center of the molten pool (Figure 3a). The molten pool interface is high on the left and low on the right, and normal angle is asymmetric at z-increment of 0.2 mm (Figure 5b).

### 3.2. Texture Evolution in Scanning Direction

**Figure 6:**
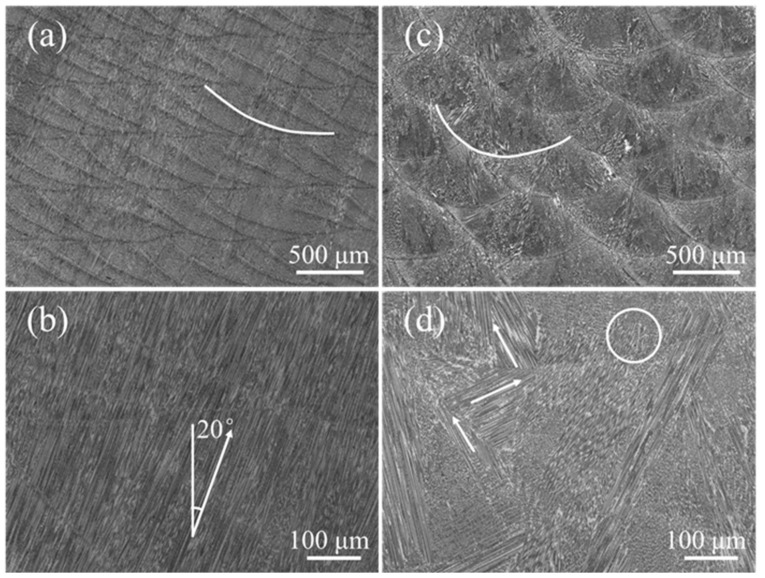
The microstructure with different pulse period: (**a**,**b**) 30 ms; (**c**,**d**) 100 ms.

Figure 6 shows optical morphologies of the longitudinal section under different pulse periods. The molten pool boundaries are clearly distinguishable and arranged regularly, but the molten pool morphology is obviously different (Figure 6a,c). The molten pool morphology is like half of a crescent, and the horizontal distance between two molten pools is 300 μm at a pulse period of 30 ms (Figure 6a), while the molten pool morphology is like fish-scale, and the horizontal distance between two molten pools is 800 μm at a pulse period of 100 ms (Figure 6c). In fact, the distance between two pulses is equal to the product of the pulse period and the scanning speed. It is worth noting that the spatial arrangement of each pulsed molten pool affects the molten pool interface. In the short pulse period, more than half of the molten pool is remelted, and a small part on the left of the molten pool is retained (Figure 6a). However, the entire bottom of the molten pool is retained in the long pulse period. Compared with continuous LAM, pulsed LAM increases the overlap between the molten pools along the scanning direction (SD).

The pulse period directly affects the epitaxial growth characteristics of dendrites in the scanning direction. A large number of dendrites grow along one direction, and the angle between the growth direction of the dendrites and BD axis is 20° at pulse period of 30 ms (Figure 6b). The direction of heat flux between different molten pulse pools is close due to the small distance [26], which is favorable for the direct epitaxial growth of columnar dendrites (Figure 6c). For a pulse period of 100 ms, no columnar dendrites were epitaxially grown across the layers (Figure 6d). There are two possible reasons for the weak epitaxial growth of columnar dendrites. The first one is large deviation of heat flow direction between adjacent molten pools due to the arc-shaped molten pool. The second one is due to the long distance of dendrite growth because of a far distance between the pulse molten pool. The zigzag dendrite marked with the white arrow appears at the edge of the molten pool (Figure 6d). It is worth noting that the dendrites marked with white circles are easily terminated near the molten pool interface in the adjacent pulsed molten pool.

**Figure 7:**
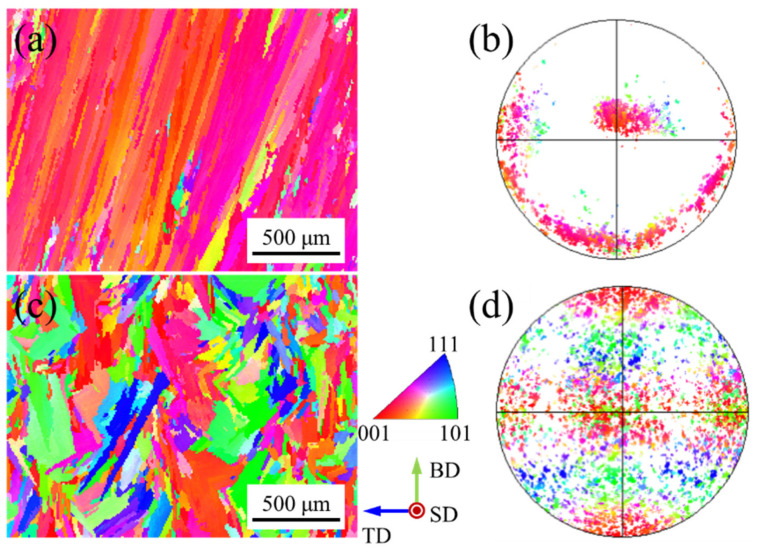
Inverse pole figure (IPF) maps and corresponding pole figure with different pulse period: (**a**,**b**) 30 ms; (**c**,**d**) 100 ms.

Figure 7 shows the inverse pole figure (IPF) map and corresponding pole figure with different pulse periods. There are many near-red grains (<100> // BD) distributed in the inverse pole figure (IPF) map at a pulse period of 30 ms (Figure 7a). In addition, fine grains of other colors are scattered. The red grains (<100> // BD) exhibit fiber texture at the corresponding pole figure (Figure 7b). When the pulse period increases, the color of the grains in the inverse pole figure (IPF) map becomes colorful at a pulse period of 100 ms (Figure 7c). The grains of the same color do not grow continuously across the layers, which indicates that the epitaxial growth of the grains is weak. The distribution of the pole point is chaotic in the corresponding pole figure (Figure 7d).

**Figure 8:**
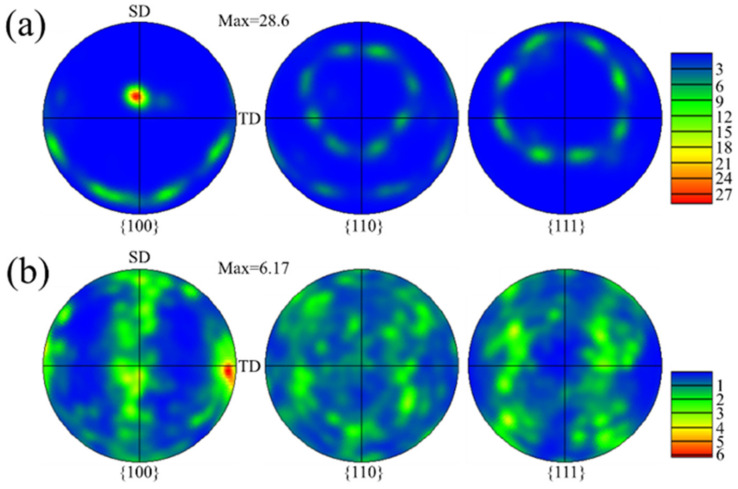
Pole figure with different pulse period: (**a**) 30 ms; (**b**) 100 ms.

Figure 8 shows the pole figures with different pulse periods. The strong fiber texture was formed with a maximum MUD of 28.6 at a pulse period of 30 ms (Figure 8a), as high as the texture intensity obtained by Wang et al. [27] using a flat-top large rectangular laser. It is worth mentioning that the position of fixed pole point (Figure 8a) is consistent with the pole position of red grains (<100> // BD) in the center of the molten pool (Figure 3b). The angle between the direction of fixed pole point and Z axis is about 19.1°. The distribution of the pole point is chaotic in the (100) pole figure at a pulse period of 100 ms, and the texture intensity is slightly weakened with a maximum MUD of 6.17 (Figure 8b). A strong pole point is located on the TD-axis, which represents the horizontal growth of dendrites at the overlapping zone of the molten pool.

**Figure 9:**
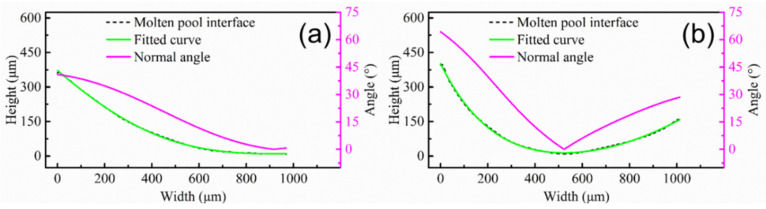
Molten pool interface and normal angle with different pulse period: (**a**) 30 ms; (**b**) 100 ms.

Figure 9 shows the molten pool interface and normal angle under different pulse periods. The interface of the molten pool is gentle, gradually decreasing from the left to the right at a pulse period of 30 ms (Figure 9a). There is a gentle curve on the right side of the molten pool. The normal angle gradually decreases from 40° on the left, and the average of the normal angle is 19.5° (Figure 9a), which is consistent with the dendrite growth direction in Figure 6b. It is worth mentioning that, even if the maximum angle between the dendrite growth direction and the value of normal angle is 20.5°, the dendrite are epitaxially grown directly at the molten pool interface. Since all dendrites are grown directly along one direction, the fiber texture is formed (Figure 8a). The molten pool interface is a curved line, and it is high on the left and low on the right at pulse period of 100 ms (Figure 9b). The normal angle first decreases and then increases (Figure 9b), which is consistent with the transverse section (Figure 5a). The pulsed LAM process is the accumulation of individual melt pools one by one. If the normal angle varies greatly at different positions, dendrites are not easy to grow epitaxially. The dendrites in the overlapping zone have epitaxial growth along secondary dendrite arms (Figure 6d). However, the difference value of normal angle is far from 90°, leading to weak epitaxial growth of dendrites along the secondary dendrite arms.

### 3.3. Texture Evolution in Track Offset Direction

**Figure 10:**
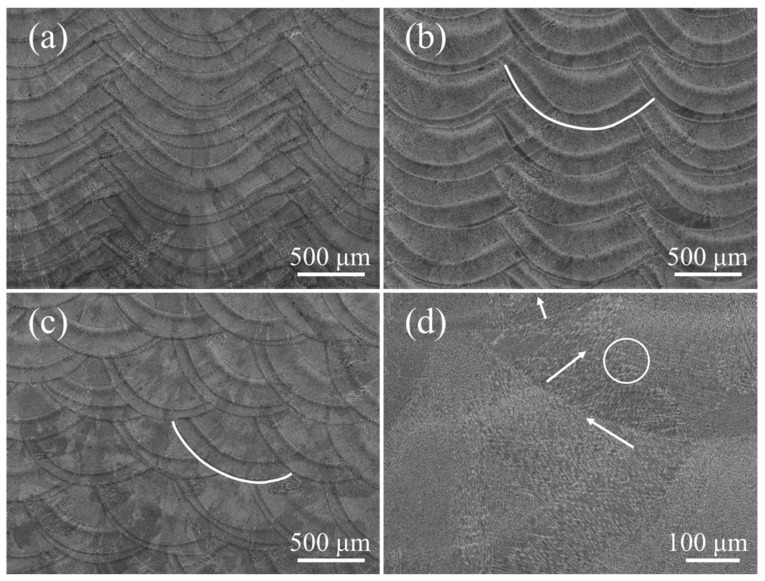
The microstructure with different track offset: (**a**) 1.4 mm; (**b**) 1 mm; (**c**,**d**) 0.6 mm.

Figure 10 shows the optical morphologies of the transverse section under different track offset. The arrangement of the molten pool is relatively regular, but the molten pool morphologies under different track offset are different. With decrease of the track offset, the molten pool morphology changes from flat to narrow and deep (Figure 10a–c). The overlapping zone is less at a track offset of 1.4 mm (Figure 10a), while the remelting area is large at a track offset of 0.6 mm (Figure 10c). The overlapping position between the molten pools is close to the center of molten pool, and the molten pools are arranged obliquely at a track offset of 0.6 mm (Figure 10c). The dendrites at the overlap of the molten pool also show a zigzag growth pattern marked with a white arrow, but the dendrites marked with a white circle are easily terminated near the molten pool interface in the adjacent pulsed melt pool (Figure 10d). The layer height for track offset of 1.4 mm, 1 mm, and 0.6 mm are 0.23 mm, 0.37 mm, and 0.47 mm, respectively. The track offset strongly affects the molten pool morphology and the molten pool spatial arrangement.

**Figure 11:**
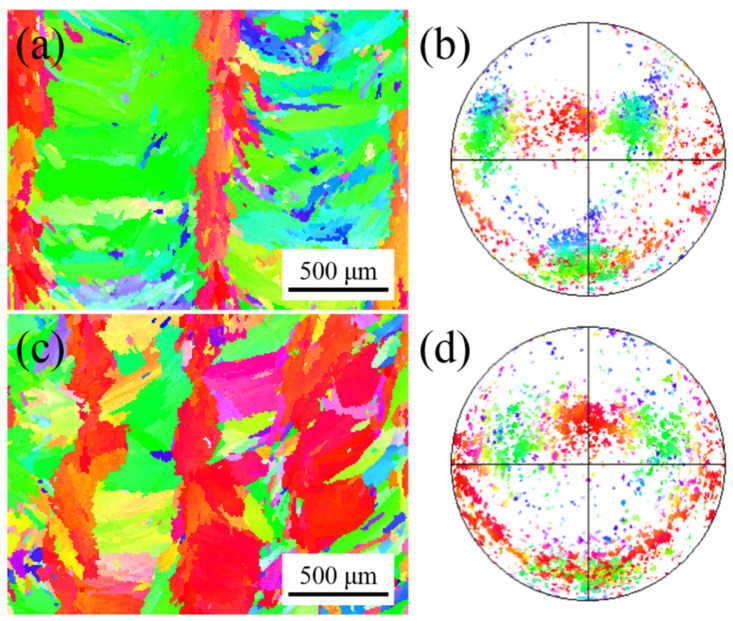
Inverse pole figure (IPF) maps and corresponding pole figure with different track offset: (**a**,**b**) 1 mm; (**c**,**d**) 0.6 mm.

Figure 11 shows the inverse pole figure (IPF) map and corresponding pole figure with different track offset. The green grains (<110> // BD) are distributed in the overlapping zones of the molten pool, while the red grains (<100> // BD) are distributed in the center of the molten pool at a track offset of 1 mm (Figure 11a). The green grains (<110> // BD) and red grains (<100> // BD) exhibit rotated cubic texture and fiber texture at the corresponding pole figure, respectively (Figure 11b), which is similar to sample B (Figure 3a). This is due to the process parameters of the two experiments being similar, as shown in Table 1. It is worth noting that the molten pool morphology is also similar (Figure 2a and Figure 10b). The red grains (<100> // BD) grown in the center of the molten pool extend to the overlapping zones, resulting in fewer green grains (<110> // BD) at a track offset of 0.6 mm (Figure 11c). The texture type has not changed, where the red grains (<100> // BD) are also fiber texture and green grains (<110> // BD) are cubic texture at the corresponding pole figure (Figure 11d).

**Figure 12:**
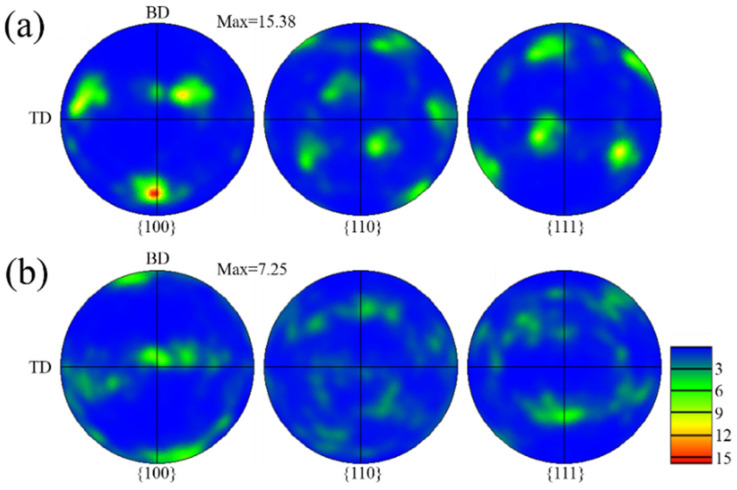
Pole figure with different track offset: (**a**) 1 mm; (**b**) 0.6 mm.

Figure 12 shows the pole figures with different track offset. Compared to the track offset of 1 mm, with a maximum MUD of 15.38 (Figure 12a), the texture intensity is decreased for the track offset of 0.6 mm with a maximum MUD of 7.25 (Figure 12b). For the track offset of 0.6 mm, the possibility of dendrites epitaxial growth along the secondary dendrite arms is weakened due to the change of the molten pool morphology and the overlapping position. The columnar dendrites are also not easy to epitaxially grow along the primary dendrite arms, because the heat flow direction is also far from the direction of the primary dendrite arms, leading to weak texture. The distribution of fiber texture is clearly visible in the (110) pole figure, which is due to the large number of red grains (<100> // BD) distributed in the inverse pole figure (IPF) map (Figure 11b).

**Figure 13:**
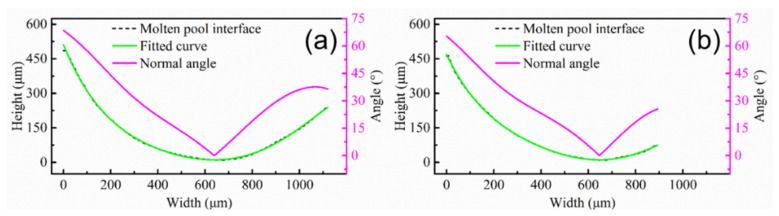
Molten pool interface and normal angle with different track offset: (**a**) 1 mm; (**b**) 0.6 mm.

Figure 13 shows the molten pool interface and normal angle under different track offset. The molten pool interface is high on the left and low on the right at a track offset of 1 mm (Figure 13a), which is similar to sample B (Figure 5b). For a track offset of 0.6 mm, the molten pool interface and the normal angle on the left are approximately same as the sample with a track offset of 1 mm, while the molten pool interface on the right is lower and the normal angle becomes smaller due to an increased overlap rate (Figure 13b). At the same time, due to the small normal angle on the right side, the dendrites are not easy to grow along the secondary dendrite arms at the overlapping zones, forming weak cubic texture (Figure 12b).

### 3.4. Growth of Dendrites at the Molten Pool Interface

The epitaxial growth of dendrites at the molten pool interface is either along the primary dendrite arms or the secondary dendrite arms. For the FCC crystal structure, the same angle relative to each of the three <001> orientations is 54.7° [5]. Since the dendrites will choose the preferred orientation with the smallest angle to the heat flow direction for growth, the angle between the heat flow direction and the dendrites growth direction is less than 54.7°. However, it is worth mentioning that the dendrites can lose their competitive advantage and be eliminated during the growth process when increasing the angle between the heat flow direction and the dendrites growth direction. For growth along secondary dendrites, the optimal angle between the heat flow directions is 90°. The epitaxial growth ability of dendrites weakens when the angle changes from 90°. For arc-shaped molten pools, dendrites grow along the secondary dendrite direction at the overlapping zone, forming V-shape dendrites. Most literature reports this dendrite growth [17,19,28].

The dendrites growth is related to the angle (θ) that is the difference value between the normal angles of the adjacent interfaces at the overlapping zone, as shown in Table 2. The θ for sample A and sample B are 86.2° and 91.1°, which are close to 90°. Additionally, the dendrites grow along the direction of the secondary dendrite arms, forming strong V-shaped grain in the overlapping zones (Figure 3a,c). It is worth noting that, since the θ of sample B is closer to 90°, the texture intensity of sample B (Figure 4b) is higher than sample A (Figure 4a). The θ for sample D and sample G are 87.8° and 65.1°, respectively. Since the θ of sample D is close to 90°, the dendrites also have epitaxial growth along the secondary dendrite arms. Thus, the texture characteristics are close to sample A and sample B (Figure 11a). However, the θ of sample G is 65.1° due to high overlap rate (Figure 13b), which is much lower than 90°. The dendrites are not prone to epitaxial growth along the secondary dendrite arms; thus, the green V-shaped grains in the overlapping zone in the inverse pole figure (IPF) map are reduced (Figure 11c) and the texture intensity in the pole figure is also reduced (Figure 12b). The molten pool interface is semicircular in the E sample (Figure 6c), and the θ is 71.83°, which is much lower than 90°. The epitaxial growth of dendrites along the secondary dendrite arms at the interface is not strong. It is worth mentioning that, compared to sample G, θ of sample E is closer to 90°, but the grain distribution of sample E (Figure 7c) is more chaotic than sample G (Figure 11c). This is because the alignment of the molten pool in the BD direction is not aligned (Figure 6c). It is difficult for dendrites to grow epitaxially between layers.

## 4. Conclusions

Inconel 718 samples were fabricated by LAM using different z-increment, pulse period and track offset. The epitaxial growth of dendrites and texture evolution in three directions were studied. The growth and competition of dendrites at the molten pool interface is discussed. A specific focus was put on the effect of molten pool spatial arrangement on the texture. Since texture affects the physical properties of the material, we can improve the performance of the material by customizing the texture. The following conclusions can be drawn:

1. The z-increment affects the formability of the formed parts. Both green grains (<110> // BD) with rotated cube texture in the molten pool overlapping zones and red grains (<100> // BD) with fiber texture in the molten pool center zones coexist, forming the typical sandwich texture feature.
2. The molten pool morphology and dendrite growth are affected by the pulse period. In a short pulse period, the dendrites can grow directly epitaxially, forming the strong fiber texture due to gentle interface and short distance. In a long pulse period, the ability of columnar dendrite epitaxial growth is weakened due to the arc-shaped molten pool, forming the weak texture.
3. With the decrease of the track offset, the molten pool morphology changes from flat to narrow and deep. For a track offset of 0.6 mm, the red grains (<100> // BD) grown in the center of the molten pool extend to the overlapping zones, resulting in fewer green grains (<110> // BD).
4. The dendrites growth is directly related to the difference value (θ) between the normal angles. When θ is close to 90°, dendrites grow along the secondary dendrite direction at the overlapping zone, forming V-shape dendrites.

## Figures and Tables

**Table 1:** Processing parameters of LAM.

Sample No	Scanning Speed (mm/s)	Pulse Period (ms)	Powder Feeding Rate (mm)	Track Offset (mm)	z-Increment (mm)
A	10	30	12	1	0.2
B	10	30	12	1	0.4
C	10	30	12	1	0.6
D	10	30	10	1	0.35
E	8	100	10	1	0.35
F	10	30	10	1.4	0.35
G	10	30	10	0.6	0.35

**Table 2:** The θ value in the overlapping zone of the molten pool.

Sample No	A	B	C	D	G
θ (°)	86.2	91.1	71.83	87.8	65.1

## Data Availability

Data sharing is not applicable.

## References

[1] DebRoy T., Wei H.L., Zuback J.S., Mukherjee T., Elmer J.W., Milewski J.O., Beese A.M., Wilson-Heid A., De A., Zhang W. (2018). Additive manufacturing of metallic components–Process, structure and properties. Prog. Mater. Sci..

[2] Collazo A., Figueroa R., Pérez C., Nóvoa X.R. (2022). Effect of Laser Speed and Hatch Spacing on the Corrosion Behavior of 316L Stainless Steel Produced by Selective Laser Melting. Materials.

[3] Ni M., Chen C., Wang X., Wang P., Li R., Zhang X., Zhou K. (2017). Anisotropic tensile behavior of in situ precipitation strengthened Inconel 718 fabricated by additive manufacturing. Mater. Sci. Eng. A.

[4] Dinda G., Dasgupta A., Mazumder J. (2012). Texture control during laser deposition of nickel-based superalloy. Scr. Mater..

[5] Chen Z.W., Guraya T., Singamneni S., Phan M.A.L. (2020). Grain Growth During Keyhole Mode Pulsed Laser Powder Bed Fusion of IN738LC. JOM.

[6] Hibino S., Todo T., Ishimoto T., Gokcekaya O., Koizumi Y., Igashira K., Nakano T. (2021). Control of Crystallographic Texture and Mechanical Properties of Hastelloy-X via Laser Powder Bed Fusion. Crystals.

[7] Calandri M., Yin S., Aldwell B., Calignano F., Lupoi R., Ugues D. (2019). Texture and Microstructural Features at Different Length Scales in Inconel 718 Produced by Selective Laser Melting. Materials.

[8] Gäumann M., Bezençon C., Canalis P., Kurz W. (2001). Single-crystal laser deposition of superalloys: Processing–microstructure maps. Acta Mater..

[9] Wang L., Wang N. (2016). Effect of substrate orientation on the formation of equiaxed stray grains in laser surface remelted single crystal superalloys: Experimental investigation. Acta Mater..

[10] Wang L., Wang N., Yao W., Zheng Y. (2015). Effect of substrate orientation on the columnar-to-equiaxed transition in laser surface remelted single crystal superalloys. Acta Mater..

[11] Liu Z., Qi H. (2015). Effects of substrate crystallographic orientations on crystal growth and microstructure formation in laser powder deposition of nickel-based superalloy. Acta Mater..

[12] Liu Z., Qi H., Jiang L. (2016). Control of crystal orientation and continuous growth through inclination of coaxial nozzle in laser powder deposition of single-crystal superalloy. J. Mater. Process. Technol..

[13] Liu Z., Qi H. (2015). Effects of processing parameters on crystal growth and microstructure formation in laser powder deposition of single-crystal superalloy. J. Mater. Process. Technol..

[14] Basak A., Das S. (2016). Epitaxy and Microstructure Evolution in Metal Additive Manufacturing. Annu. Rev. Mater. Res..

[15] Takaki T., Ohno M., Shibuta Y., Sakane S., Shimokawabe T., Aoki T. (2016). Two-dimensional phase-field study of competitive grain growth during directional solidification of polycrystalline binary alloy. J. Cryst. Growth.

[16] Garibaldi M., Ashcroft I., Simonelli M., Hague R. (2016). Metallurgy of high-silicon steel parts produced using Selective Laser Melting. Acta Mater..

[17] Sun S.-H., Ishimoto T., Hagihara K., Tsutsumi Y., Hanawa T., Nakano T. (2019). Excellent mechanical and corrosion properties of austenitic stainless steel with a unique crystallographic lamellar microstructure via selective laser melting. Scr. Mater..

[18] Gokcekaya O., Ishimoto T., Hibino S., Yasutomi J., Narushima T., Nakano T. (2021). Unique crystallographic texture formation in Inconel 718 by laser powder bed fusion and its effect on mechanical anisotropy. Acta Mater..

[19] Andreau O., Koutiri I., Peyre P., Penot J.-D., Saintier N., Pessard E., De Terris T., Dupuy C., Baudin T. (2019). Texture control of 316L parts by modulation of the melt pool morphology in selective laser melting. J. Mater. Process. Technol..

[20] McLouth T.D., Bean G.E., Witkin D.B., Sitzman S.D., Adams P.M., Patel D.N., Park W., Yang J.-M., Zaldivar R.J. (2018). The effect of laser focus shift on microstructural variation of Inconel 718 produced by selective laser melting. Mater. Des..

[21] Cheng M., Xiao X., Luo G., Song L. (2021). Integrated control of molten pool morphology and solidification texture by adjusting pulse duration in laser additive manufacturing of Inconel 718. Opt. Laser Technol..

[22] Xiao H., Li S., Han X., Mazumder J., Song L. (2017). Laves phase control of Inconel 718 alloy using quasi-continuous-wave laser additive manufacturing. Mater. Des..

[23] Nenadl O., Ocelík V., De Hosson J.T.M. (2017). Texture development in direct powder deposition. J. Laser Appl..

[24] Wang Y., Yu C., Xing L., Li K., Chen J., Liu W., Ma J., Shen Z. (2020). Grain structure and texture of the SLM single track. J. Mater. Process. Technol..

[25] Luo G., Xiao H., Li S., Wang C., Zhu Q., Song L. (2019). Quasi-continuous-wave laser surface melting of aluminium alloy: Precipitate morphology, solute segregation and corrosion resistance. Corros. Sci..

[26] Liu X., Xiao H., Xiao W., Song L. (2021). Microstructure and Crystallographic Texture of Laser Additive Manufactured Nickel-Based Superalloys with Different Scanning Strategies. Crystals.

[27] Wang Y., Shi J. (2020). Developing very strong texture in a nickel-based superalloy by selective laser melting with an ultra-high power and flat-top laser beam. Mater. Charact..

[28] Wan H., Zhou Z., Li C., Chen G., Zhang G. (2018). Effect of scanning strategy on grain structure and crystallographic texture of Inconel 718 processed by selective laser melting. J. Mater. Sci. Technol..

